# Rhinovirus Infection Is Associated With Airway Epithelial Cell Necrosis and Inflammation via Interleukin-1 in Young Children With Cystic Fibrosis

**DOI:** 10.3389/fimmu.2020.00596

**Published:** 2020-04-09

**Authors:** Samuel T. Montgomery, Dario L. Frey, Marcus A. Mall, Stephen M. Stick, Anthony Kicic, AREST CF

**Affiliations:** ^1^Faculty of Health and Medical Sciences, School of Biomedical Sciences, The University of Western Australia, Crawley, WA, Australia; ^2^Department of Translational Pulmonology, Translational Lung Research Center Heidelberg, University of Heidelberg, Heidelberg, Germany; ^3^German Center for Lung Research, Heidelberg, Germany; ^4^Department of Pediatric Pulmonology, Immunology and Critical Care Medicine, Charité-Universitätsmedizin Berlin, Berlin, Germany; ^5^Berlin Institute of Health, Berlin, Germany; ^6^Telethon Kids Institute, The University of Western Australia, Crawley, WA, Australia; ^7^Department of Respiratory and Sleep Medicine, Perth Children's Hospital, Nedlands, WA, Australia; ^8^Centre for Cell Therapy and Regenerative Medicine, School of Medicine and Pharmacology, The University of Western Australia, Nedlands, WA, Australia; ^9^School of Public Health, Curtin University, Bentley, WA, Australia; ^10^St John of God Hospital, Subiaco, WA, Australia; ^11^Murdoch Children's Research Institute, Melbourne, VIC, Australia; ^12^Department of Paediatrics, University of Melbourne, Melbourne, VIC, Australia

**Keywords:** cystic fibrosis, airway epithelium, rhinovirus, interleukin-1, necrosis

## Abstract

**Introduction:** The responses of cystic fibrosis (CF) airway epithelial cells (AEC) to rhinovirus (RV) infection are likely to contribute to early pathobiology of lung disease with increased neutrophilic inflammation and lower apoptosis reported. Necrosis of AEC resulting in airway inflammation driven by IL-1 signaling is a characteristic finding in CF detectable in airways of young children. Being the most common early-life infection, RV-induced epithelial necrosis may contribute to early neutrophilic inflammation in CF via IL-1 signaling. As little is known about IL-1 and biology of CF lung disease, this study assessed cellular and pro-inflammatory responses of CF and non-CF AEC following RV infection, with the hypothesis that RV infection drives epithelial necrosis and IL-1 driven inflammation.

**Methods:**Primary AEC obtained from children with (*n* = 6) and without CF (*n* = 6) were infected with RV (MOI 3) for 24 h and viable, necrotic and apoptotic events quantified via flow cytometry using a seven-step gating strategy (% total events). IL-1α, IL-1β, IL-1Ra, IL-8, CXCL10, CCL5, IFN-β, IL-28A, IL-28B, and IL-29 were also measured in cell culture supernatants (pg/mL).

**Results:**RV infection reduced viable events in non-CF AEC (*p* < 0.05), increased necrotic events in non-CF and CF AEC (*p* < 0.05) and increased apoptotic events in non-CF AEC (*p* < 0.05). Infection induced IL-1α and IL-1β production in both phenotypes (*p* < 0.05) but only correlated with necrosis (IL-1α: *r* = 0.80; IL-1β: *r* = 0.77; *p* < 0.0001) in CF AEC. RV infection also increased IL-1Ra in non-CF and CF AEC (*p* < 0.05), although significantly more in non-CF AEC (*p* < 0.05). Finally, infection stimulated IL-8 production in non-CF and CF AEC (*p* < 0.05) and correlated with IL-1α (*r* = 0.63 & *r* = 0.74 respectively; *p* < 0.0001).

**Conclusions:**This study found RV infection drives necrotic cell death in CF AEC. Furthermore, RV induced IL-1 strongly correlated with necrotic cell death in these cells. As IL-1R signaling drives airway neutrophilia and mucin production, these observations suggest RV infection early in life may exacerbate inflammation and mucin accumulation driving early CF lung disease. Since IL-1R can be targeted therapeutically with IL-1Ra, these data suggest a new anti-inflammatory therapeutic approach targeting downstream effects of IL-1R signaling to mitigate viral-induced, muco-inflammatory triggers of early lung disease.

## Introduction

Cystic Fibrosis (CF) lung disease is progressive, evolves within the first months of life, and is characterized by mucus obstruction and inflammation observable on CT even in the absence of clinical symptoms and often in the absence of detectable respiratory infection (1, 2). Neutrophilic inflammation is a key risk factor for airway disease resulting in bronchiectasis and loss of lung function (3). However, the link between mucus obstruction and airway inflammation has not yet been clearly identified.

Recent evidence from the Australian Respiratory Early Surveillance Team for CF (AREST CF) implicates mucin accumulation as the initial trigger of neutrophilic inflammation in the CF airway (4), and suggests respiratory viral infection may trigger the muco-inflammatory phenotype observed in CF since the heterogeneity of early CF lung disease mirrors the heterogeneity of childhood viral infection (5, 6). Human rhinovirus (RV) appears to be able to manipulate host responses switching from apoptotic to necrotic cell death in airway epithelial cells (AEC) (7, 8). Studies investigating non-bacterial inflammation in the CF airway microenvironment have linked interleukin (IL)-1R signaling driven by IL-1α released from necrotic AEC to neutrophilic inflammation (9, 10). As RV is the most common early life viral infection observed in children with CF (11) and IL-1R signaling has already been detected in the airways of young children with mild disease (12), we hypothesize that resultant neutrophilic inflammation may be driven via this signaling pathway triggered by RV-induced AEC necrosis. However, this proposed mechanism has yet to be investigated.

Given our previous observations of defective responses to RV (8) and IL-1 driven inflammatory responses to necrosis in the pediatric CF airway (12), this study aimed to investigate the direct relationship between RV infection, the type of induced cell death, and IL-1R-driven inflammation *in vitro* using primary AEC from infants and young children with CF. We obtained primary AEC from young children with and without CF and assessed viable, necrotic and apoptotic events following RV infection utilizing flow cytometry. Using experimental supernatants; IL-1α, IL-1β, IL-1Ra, sIL-1R2, IL-8, CXCL10, CCL5, IFN-β, IL-28A, IL-28B, and IL-29 were measured and subsequently correlated to viable, necrotic and apoptotic responses.

## Materials and Methods

Please also refer to the Supplementary Data for full details.

### Study Population and Establishment of Primary Cell Culture

This study was approved by the relevant institutional Human Ethics Committees with written consent obtained from parents or guardians. This study included samples from six clinically stable infants and children with CF (mean age 2.9 ± 1.8 years old; Table 1) participating in the AREST CF early surveillance program (2), and samples from six children without CF (mean age 3.8 ± 1.9 years old; Table 1) recruited upon admission to hospital for elective non-respiratory related surgery. Cystic fibrosis transmembrane conductance regulator (CFTR) genotype was determined as part of newborn screening (Table 1). Current bacterial infection in CF samples was determined as part of standard clinical practice using gold-standard microbiological screening, with previous infection the presence of a bacterial infection at any previous visit. Prior wheeze was determined by parent-reported wheeze in the three-months prior to recruitment. Children without CF had no respiratory symptoms observed at time of recruitment. Samples were attained by brushing of the tracheal mucosa of children with a single-sheathed nylon bronchial cytology brush as previously described (8, 13). After collection, primary AEC cultures were established as previously described (14).

**Table 1:** Demographics of the study population.

	**Non-CF**	**CF**
Number of subjects	6	6
Age (mean ± standard deviation)	3.52 ± 1.6 years	3.16 ± 0.98 years
Sex (% Male)	66.6%	50%
Prior wheeze status (% Wheeze)	50%	0%
Genotype (% Phe.508del homozygous)	N/A	83.3%
Current bacterial infection	N/A	50%
Previous bacterial infection	N/A	33.3%
Neutrophil elastase presence	N/A	33.3%

### Human RV Infection

Human rhinovirus 1b (RV1b) was propagated as previously described (15). To simulate an acute RV infection *in vitro*, primary AEC were infected with ~2.95 × 10^5^ TCID_50_/mL. To ensure responses were due to actively replicating virus, controls were exposed to an UV-inactivated RV1b at the same TCID_50_ as previously described (16). After 24- and 48-h cells were collected for analysis via flow cytometry and supernatant collected for cytokine measurement. As the peak concentration of RV viral load following infection is observed 24 h post-infection (17, 18), this timepoint was chosen for analysis. Viral load was assessed via qPCR as previously described (19). Infection with RV1b induced typical viral cytokine production from both non-CF and CF AEC (Table S1). Data from 48 h of RV1b infection is presented in the Supplementary Data.

### Flow Cytometry

A flow cytometry methodology to measure cell death and disassembly was adapted for use with AEC (20). Briefly, primary cells were detached from culture surfaces via gentle trypsinization, combined with cells obtained from supernatant following centrifugation, and resuspended at a concentration of 10^6^ cells/mL in annexin binding buffer (ThermoFisher Scientific, Scoresby, VIC, Australia). Tubes containing 100 μL of cell suspension were stained for 15 min with 100 μL of Annexin V/AlexaFluor488 (ThermoFisher Scientific, Scoresby, VIC, Australia) (1:40 v/v) and TO-PRO-3 (10 μM final concentration) (ThermoFisher Scientific, Scoresby, VIC, Australia) in annexin binding buffer and flow cytometry performed via a FACSCanto II flow cytometer (BD Biosciences, Franklin Lakes, NJ, USA). A total of 20,000 events were recorded during acquisition for each sample. Analysis was performed using FlowJo software v10.4 (FlowJo LLC, Ashland, OR, USA) using a seven-step gating strategy to separate events into viable, necrotic, A5+ apoptotic, A5- apoptotic, apoptotic bodies and cellular debris as previously described (20) (Figure S1). Cutoffs used for positive forward scatter (FSC) and side scatter (SSC) were 50 k. Events were grouped into “viable”, “necrotic”, and “apoptotic” for further analysis. Data are presented as percentage of total events (% total).

### Cytokine Measurement

Interleukin (IL)-1α, IL-1β, and interferon- beta (IFN-β) protein production was determined using commercially available AlphaLISA kits (Perkin Elmer, Waltham, MA, USA) in cell-free culture supernatant. Similarly, IL-8 (BD Biosciences, San Diego, CA, USA), IL-1 receptor antagonist (IL-1Ra), soluble IL-1 receptor 2 (sIL-1R2), C-X-C motif chemokine 10 (CXCL10), Chemokine (C-C motif) ligand 5 (CCL5), IL-28A, IL-28B, and IL-29 protein production (R&D Systems, Minneapolis, MN, USA) were all determined using commercially available ELISA kits performed according to manufacturer's instructions. Samples below the detection range were arbitrarily reported as half the lower limit and included in the analysis with all other samples as previously described (21).

### Statistical Analysis

Data were analyzed using GraphPad Prism v7.04 (GraphPad Software, La Jolla, CA, USA). Data were natural log transformed where appropriate. Comparisons between paired data were performed using Wilcoxon matched pairs signed rank test and Friedman's test with Dunn's multiple comparisons test presented as mean ± standard deviation. Comparisons between unpaired data were performed using Mann-Whitney tests presented as mean ± standard deviation. Associations between flow cytometry events and cytokines measured were assessed using Spearman's rank-order correlations. A two tailed *P* value < 0.05 was considered statistically significant.

## Results

Demographic data for the study populations are summarized in Table 1. Sex and age were similar between cohorts, with most children with CF homozygous for the p.Phe508del mutation. Infection with RV1b resulted in increased rhinovirus load measured via qPCR compared to UV-inactivated RV1b (31.3 ± 29.8 copy #/ng RNA vs. 2.37 × 10^7^ ± 1.46 × 10^7^ copy #/ng RNA; *p* < 0.05), increased typical pro-inflammatory viral cytokines CXCL10 and CCL5 (Figure S2), and type I and III interferon responses (Figures S3, S4).

### Rhinovirus Infection Increases Necrosis but Not Apoptosis in CF AEC

**Figure 1:**
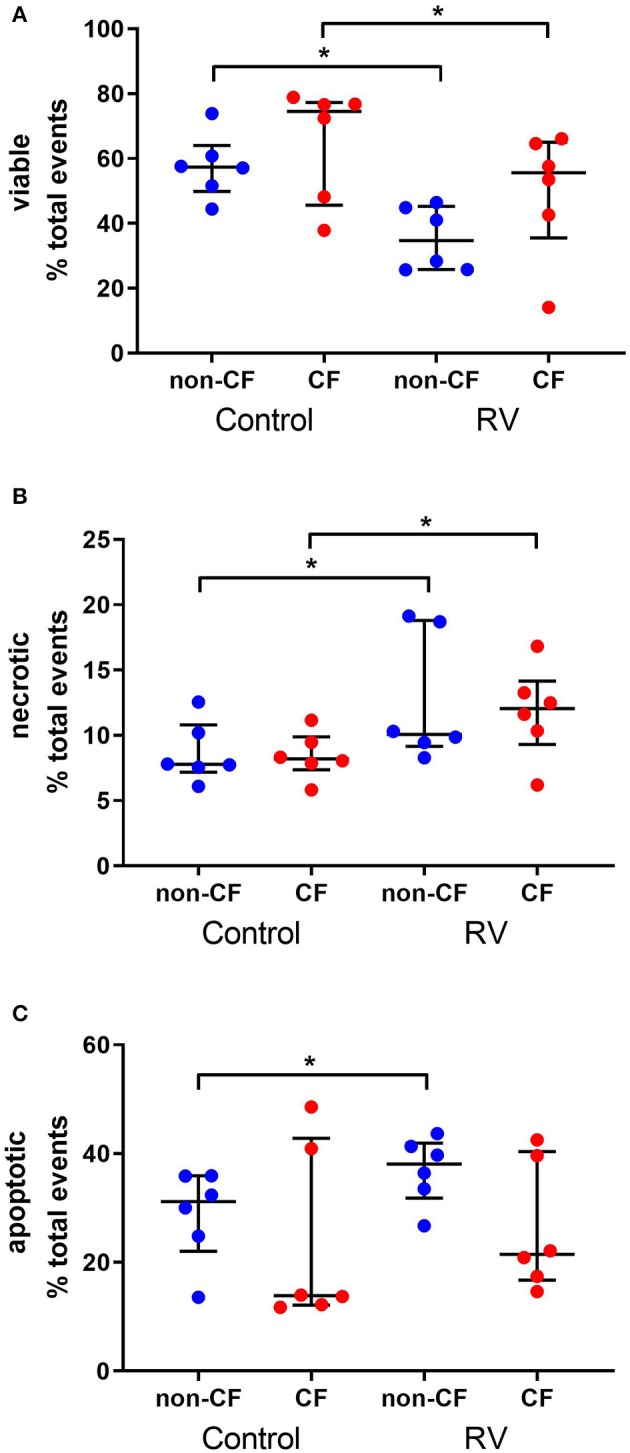
Rhinovirus infection of non-CF and CF AEC decreases viable events, increases necrotic events, and increases apoptotic events in non-CF AEC only. Non-CF (*n* = 6) and CF (*n* = 6) AEC infected with RV1b for 24 h were assessed for changes in viable **(A)**, necrotic **(B)**, and apoptotic **(C)** events measured via flow cytometry. Infection with RV1b for 24 h resulted in **(A)** decreased viable events in non-CF and CF AEC compared to controls, **(B)** increased necrotic events in non-CF and CF AEC compared to controls, and **(C)** increased apoptotic events in non-CF AEC compared to controls. *p < 0.05.

To determine the cellular response to rhinovirus infection, we measured viable, necrotic, and apoptotic events in non-CF (*n* = 6) and CF (*n* = 6) AEC (Figure 1). Infection with RV1b resulted in reduced viable events in non-CF AEC (57.6 ± 9.8% vs. 35.4 ± 9.8%; *p* < 0.05) and CF AEC (65.1 ± 17.5% vs. 49.8 ± 19.4%; *p* < 0.05) (Figure 1A), and significantly elevated necrotic events in non-CF AEC (8.7 ± 2.3% vs. 12.6 ± 4.9%; *p* < 0.05) and CF AEC (8.5 ± 1.8% vs. 11.8 ± 3.5%; *p* < 0.05) (Figure 1B). RV1b infection significantly increased apoptotic events in non-CF AEC (28.8 ± 8.5% vs. 36.9 ± 6.1%; *p* < 0.05), however, this was not observed for CF AEC (23.5 ± 16.6% vs. 26.2 ± 11.9%) (Figure 1C). Similarly, infection with RV1b for 48 h decreased viable events, increased necrotic events, and increased apoptotic events in both non-CF and CF AEC (Figure S5).

### IL-1α and IL-1β Are Increased in Supernatant and Correlate With Cell Death Following Rhinovirus Infection

**Figure 2:**
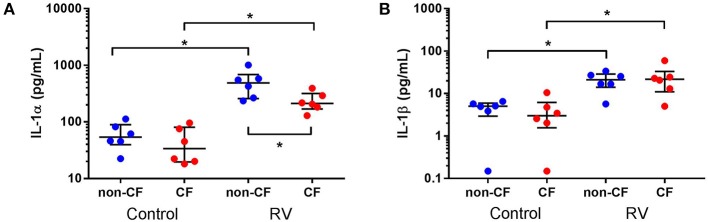
IL-1α and IL-1β is increased in supernatant from non-CF and CF AEC following rhinovirus infection. Supernatant from non-CF (*n* = 6) and CF (*n* = 6) AEC infected with RV1b at for 24 h was assessed for levels of IL-1α and IL-1β protein. Infection with RV1b for 24 h resulted in **(A)** increased IL-1α from non-CF and CF AEC compared to control, with higher levels in non-CF supernatant compared to CF supernatant post-infection, and **(B)** increased IL-1β from non-CF and CF AEC compared to controls. *p < 0.05.

**Figure 3:**
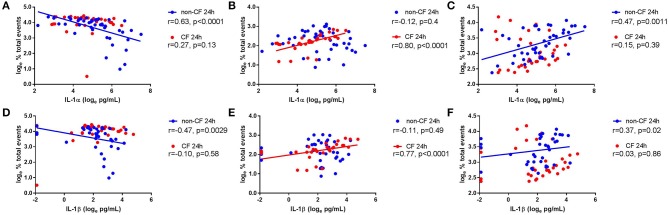
IL-1α and IL-1β in supernatant are associated with necrotic events in CF AEC but not non-CF AEC following 24 h of rhinovirus infection. IL-1α and IL-1β protein in supernatant from non-CF (*n* = 44) and CF (*n* = 32) AEC following RV1b infection for 24 h were assessed for correlations with the corresponding changes in viable, necrotic and apoptotic events measured via flow cytometry. IL-1α protein in supernatant was **(A)** significantly correlated with decreased viable events in non-CF AEC but not CF AEC, **(B)** significantly correlated with increased necrotic events in CF AEC but not non-CF AEC, and **(C)** significantly correlated with increased apoptotic events in non-CF AEC but not CF AEC. Similarly, IL-1β protein in supernatant was **(D)** significantly correlated with decreased viable events in non-CF AEC but not CF AEC, **(E)** significantly correlated with increased necrotic events in CF AEC but not non-CF AEC, and **(F)** significantly correlated with increased apoptotic events in non-CF AEC but not CF AEC.

We next investigated the role of IL-1 signaling in the inflammatory response following rhinovirus-induced cell death *in vitro* by measuring IL-1α and IL-1β protein following RV1b infection and correlated these with viable, necrotic, and apoptotic events in non-CF and CF AEC (Figure 2). Infection with RV1b increased IL-1α in non-CF (61.6 ± 31.7 pg/mL vs. 511 ± 252 pg/mL; *p* < 0.05) and CF AEC supernatant compared to controls (46.2 ± 32.7 pg/mL vs. 236 ± 93.1 pg/mL; *p* < 0.05) (Figure 2A). IL-1α was higher in supernatant from non-CF AEC when compared to CF AEC (*p* < 0.05). Similarly, IL-1β protein was significantly elevated post infection in both non-CF (4.4 ± 2.3 pg/mL vs. 20.9 ± 9.9 pg/mL; *p* < 0.05) and CF AEC (3.9 ± 3.6 pg/mL vs. 24.2 ± 18.7 pg/mL; *p* < 0.05) (Figure 2B) supernatant when compared to controls. Upon analysis, IL-1α was found to be negatively correlated with viable events measured in non-CF AEC only (*r* = −0.63, *p* < 0.0001), positively correlated with necrotic events measured in CF AEC (*r* = 0.80, *p* < 0.0001), as well as apoptotic events measured in non-CF (*r* = 0.47, *p* = 0.0011) (Figures 3A–C). Similarly, IL-1β was negatively correlated with viable events measured in non-CF (*r* = −0.47, *p* = 0.0029), strongly positively correlated with necrotic events measured in CF AEC (*r* = 0.77, *p* < 0.0001). A weak correlation was also observed between IL-1β and apoptotic events measured in non-CF AEC only (*r* = 0.37, *p* < 0.05) (Figures 3D–F). Infection with RV1b for 48 h produced similar responses, with increased IL-1α and IL-1β following infection (Figure S6) significantly associated with necrotic events only in CF AEC, but with apoptotic events in non-CF and CF AEC (Figure S7).

### IL-1Ra Is Increased in Supernatant Following Rhinovirus Infection

**Figure 4:**
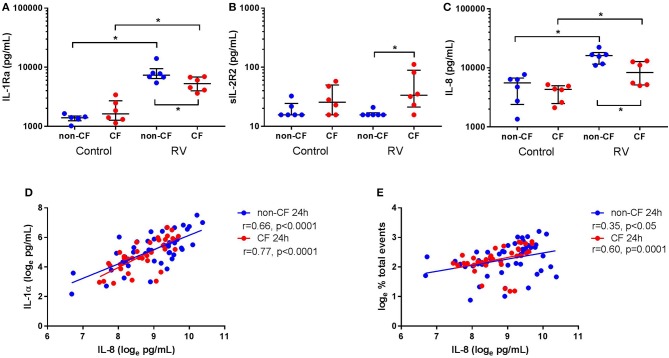
Rhinovirus infection increases IL-1Ra and IL-8 signaling in supernatant from non-CF and CF AEC. Supernatant from non-CF (*n* = 6) and CF (*n* = 6) AEC infected with RV1b for 24 h was assessed for levels of IL-1Ra, sIL-1R2, IL-8 protein, and correlations between IL-8 and IL-1α protein or necrotic events measured via flow cytometry. Infection with RV1b for 24 h resulted in **(A)** increased IL-1Ra from non-CF and CF AEC compared to control, with significantly higher IL-1Ra in supernatant from non-CF AEC compared to CF AEC, **(B)** no change in sIL-1R2 from non-CF and CF AEC compared to control, but significantly higher sIL-1R2 after infection in supernatant from CF AEC compared to non-CF AEC, and **(C)** increased IL-8 in supernatant from non-CF and CF AEC compared to control, with significantly higher IL-8 in supernatant from non-CF AEC compared to CF AEC which **(D)** significantly correlated with IL-1α levels in supernatant from non-CF and CF AEC and **(E)** significantly correlated with increased necrotic events in both non-CF and CF AEC. *p < 0.05.

Since we observed differential responses in IL-1 signaling, we next assessed IL-1R regulatory protein expression, namely IL-1Ra and sIL-1R2, by non-CF and CF AEC following infection with RV1b (Figure 4). Rhinovirus infection resulted in increased IL-1Ra production from non-CF (1368.2 ± 205.6 pg/mL vs. 8149.0 ± 3013.1 pg/mL; *p* < 0.05) and CF AEC (1930.4 ± 870.4 pg/mL vs. 5334.1 ± 1425.4 pg/mL; *p* < 0.05) compared to control, with significantly higher IL-1Ra observed in non-CF AEC after infection compared to CF AEC (*p* < 0.05) (Figure 4A). There was no difference in sIL-1R2 protein production between non-CF or CF AEC, however, sIL-1R2 was significantly induced after infection in CF AEC when compared to non-CF AEC (16.5 ± 2.1 pg/mL vs. 49.8 ± 38.1 pg/mL; *p* < 0.05) (Figure 4B). Similarly, infection with RV1b for 48 h increased IL-1Ra but not sIL-1R2 production in both non-CF and CF AEC (Figure S8).

### IL-8 Is Increased in Supernatant and Associated With IL-1α and Necrotic Events Following Rhinovirus Infection

We next measured inflammation downstream of IL-1R activation by measuring levels of the main neutrophil chemoattractant, IL-8, by non-CF and CF AEC following RV1b infection. Viral infection resulted in a significant increase in IL-8 protein by both non-CF (4890.5 ± 2426.7 pg/mL vs. 15656.4 ± 4102.1 pg/mL; *p* < 0.05) and CF AEC (3915.3 ± 1262.1 pg/mL vs. 8762.8 ± 3919.0 pg/mL; *p* < 0.01) compared to relevant controls (Figure 4C), with significantly higher IL-8 produced by non-CF AEC compared to CF AEC (*p* < 0.05). After infection for 48 h, IL-8 was significantly increased in non-CF and CF AEC (Figure S9A). When analyzed for associations with IL-1 signaling and cell death, IL-8 was positively correlated with necrotic events in non-CF and CF AEC (*r* = 0.35, *p* < 0.05 and *r* = 0.60, *p* = 0.0001 respectively) (Figure 4D), and IL-1α in non-CF and CF AEC (*r* = 0.63 & *r* = 0.74 respectively; *p* < 0.0001) (Figure 4E). Similar responses were observed following 48 h of infection, with significant associations between IL-8 and IL-1α and necrotic events in non-CF and CF AEC (Figures S9B,C).

## Discussion

Our previous work demonstrated a defective response of CF AEC to RV infection (8), and an inflammatory response to epithelial necrosis in CF driven by IL-1R signaling (9) that is already detectable in the airways of infants and children with CF in the absence of bacterial infection (12). In the current study, we add to these earlier findings by conducting a series of *in vitro* experiments on AEC from children with and without CF focusing on the response of the epithelium to RV infection. Utilizing flow cytometry we observed increased necrosis in CF AEC associated with IL-1R signaling, but increased apoptosis in non-CF AEC associated with IL-1R signaling. When we assessed the IL-1 receptor antagonist IL-1Ra, we found that RV induced IL-Ra production in both phenotypes however this was significantly higher in non-CF AEC. This corresponded with increased IL-8 following RV infection that was significantly higher in non-CF AEC. Furthermore, production of IL-8 was associated with IL-1α and epithelial necrosis in non-CF and CF AEC.

This study provides several novel insights into the mechanisms surrounding pro-inflammatory responses and cell death following RV infection in the CF airway. Our data shows RV infection directly increases necrotic events in both non-CF and CF AEC supporting previous data where rhinovirus protease 3C increased necrosis in nasal AEC (7). The lack of apoptosis in CF AEC supports previous work in our laboratory where dampened apoptosis was observed following RV infection (8). This study supports data suggesting RV infection drives lytic cell death (7), potentially responsible for the increased viral load observed in CF (8, 22).

Delayed apoptosis was also observed in CF AEC following RV infection in this study. Defective apoptotic responses have been observed in AEC and neutrophils in CF (8, 23, 24), we hypothesize reported accumulation of apoptotic cells in the CF airway may be suggestive evidence of defective efferocytosis (25, 26). Cleavage of the phosphatidylserine receptor by neutrophil elastase specifically disrupts phagocytosis of apoptotic cells (26, 27) and as free neutrophil elastase is increased in the CF airway (28, 29), it may explain the reduced apoptotic response and defective efferocytosis observed in the CF airway. Additionally, as suggested by the data in this study, a delayed apoptotic response following RV infection of AEC may also contribute to the defective apoptosis and increased viral load observed in CF (8, 22). The study by Vandivier et al. also found evidence of secondary necrosis following delayed apoptosis, potentially further exacerbating inflammation in the airway via release of DAMPs such as IL-1 signaling (26). As phagocytosis of apoptotic cells can induce anti-inflammatory cytokine production (30, 31), impaired clearance of apoptotic cells may have an additive effect on airway inflammation via reduced anti-inflammatory capacity.

Neutrophilic inflammation is a key risk factor for airway disease resulting in bronchiectasis and loss of lung function (3) which is observed in the absence of detectable bacterial infection (1, 2, 10). It is therefore important to elucidate triggers of early inflammation prior to bacterial colonization of the CF airway. As IL-1R signaling has been investigated as a key pathway driving neutrophilic and eosinophilic inflammation in the airway (9, 12, 32, 33), we next investigated IL-1α and IL-1β signaling following RV infection of AEC. As IL-1α is constitutively active, it can be released directly from necrotic cells in the airway epithelium (9) or actively secreted following activation of the NLRP3 inflammasome and caspase-1 (34, 35) which is required for IL-1β cleavage and release. Activation of the NLRP3 inflammasome has been reported following RV infection resulting from calcium flux resulting from RV ion channel protein 2B activity (36), potassium efflux from lytic cell death such as necrosis or pyroptosis (37), and dysregulated sodium transport due to ENaC upregulation (38). It has also been observed in other inflammatory respiratory diseases with RV associated exacerbations as a hallmark of disease like asthma or COPD, where viral-induced cell death likely contributes to morbidity (36, 39, 40). In this study, we found increased IL-1α and IL-1β alongside increased necrotic cell death suggesting NLRP3 activation could potentially exacerbate the inflammatory cascade following RV infection. This finding supports previously reported data that both IL-1α and IL-1β are released from AEC following RV infection and implicated active secretion via NLRP3 activation (41, 42). Additionally, IL-1α and IL-1β in supernatants of airway mucopurulent secretions have been shown to regulate both MUC5B and MUC5AC through IL-1R (43–45). Release of IL-1α is primarily through AEC while IL-1β in the CF lung is mainly released from macrophages and interstitial mononuclear cells (46, 47), potentially explaining the differences between IL-1α and IL-1β levels observed in this study when compared to levels reported in other studies in *ex-vivo* samples (43). This data suggests IL-1α and IL-1β observed following RV-A infection may exacerbate mucus hyperconcentration and obstruction evident in the CF airway (4, 43).

Furthermore, we found IL-1α and IL-1β significantly correlated with necrotic events in CF AEC only, while IL-1α and IL-1β correlated with apoptotic events in non-CF AEC only. Studies utilizing the β-ENaC murine model of CF-like lung disease have observed the presence of mucus obstruction and airway neutrophilia in germ-free conditions (48, 49), with “sterile” inflammation in the CF airway triggered by IL-1α released from necrotic AEC (9, 50). IL-1α is measurable in the airways of young children with CF with mild lung disease and associated with structural lung disease measured via CT in the absence of detectable bacterial infection, suggesting a role for IL-1α in the inflammatory cascade in the CF airway environment in the absence of detectable bacterial infection (12). The current study observed levels of IL-1α higher than measured in BALf in young children with CF, suggesting clinically relevant amounts of IL-1α are released from AEC following RV infection. There was higher IL-1α detected in non-CF AEC compared to CF AEC in response to RV infection suggesting IL-1α release from CF AEC occurs predominantly via necrotic cell death post-infection, and release from non-CF AEC via apoptotic cell death. Additionally, IL-1α is associated with viability of non-CF AEC, suggesting overall cell death had a greater effect on IL-1α release in non-CF AEC. Apoptotic cell death is considered immunologically silent due to efficient phagocytosis (51), however, in an *in vitro* monoculture there is a lack of clearance which results in secondary necrosis and cellular breakdown (52). While epithelial cells can self-phagocytize to reduce inflammatory consequences (53, 54), clearance of apoptotic cells relies on professional phagocytes like macrophages (55) and failure leads to release of immunostimulatory danger associated molecular patterns such as IL-1α (56). Secondary necrosis of AEC *in vitro* may potentially explain the differences in IL-1α detected between phenotypes, likely due to the observed and reported lack of apoptosis in CF AEC following RV infection (8). Defective apoptosis due to cleavage of apoptotic signaling receptors by neutrophil elastase and manipulation of phagocytic ability by *Pseudomonas aeruginosa* in monocytes has been reported in CF (26, 57). As IL-1α is increased in the CF airway during bacterial infection (12), we hypothesize defective apoptotic signaling and efferocytosis may play a role in IL-1R-activated neutrophilic inflammation in the CF airway before and after bacterial colonization of the CF airway.

Several recent studies have shown the potential for anti-inflammatory therapy by blocking of IL-1R via genetic deletion of the receptor or pharmacological inhibition via IL-1Ra to inhibit IL-8 expression and neutrophilic inflammation (9, 43). Deletion of IL-1R and IL-1Ra treatment in the βENaC-transgenic mouse significantly reduced IL-1β, neutrophils present in the airway and levels of keratinocyte chemoattractant—a murine IL-8 ortholog (9). This finding was also observed in primary AEC grown at air-liquid interface after stimulation with supernatants of airway mucopurulent secretions, with IL-1Ra treatment reducing IL-8 mRNA (43). The present study found increased IL-1Ra following RV infection in both non-CF and CF AEC, although IL-1Ra was higher in non-CF AEC when compared to CF AEC. This did not correspond with a reduction in IL-8 signaling likely as a result of the amount measured being dramatically lower than the therapeutic concentrations used in other studies (9, 43). RV infection increased IL-8 in both non-CF and CF AEC, however it was significantly higher in non-CF AEC post-infection. This contrasts with previous data by Sutanto et al. which demonstrated significantly higher IL-8 from CF AEC post-RV infection (8). However, differences in the viral titer used for infection and shorter timepoint may have contributed toward the differences in the observed findings.

There are number of unique strengths to the current study. Firstly, primary AEC from pediatric patients were used for experiments in this study, as most immortalized cell lines that are commonly used in CF research are derived from adult donors and may not accurately recapitulate phenotypic differences observed following RV infection in primary AEC isolated from the pediatric airway (8). Secondly, primary cell cultures were passaged before use in this study to distance *in vitro* cultures from the inflammatory environment from which they were isolated to minimize any pro-inflammatory influences from the *in vivo* airway milieu (58). While using freshly isolated AEC for *in vitro* studies may more accurately recapitulate the environment in the CF airway, it could obfuscate mild and virus-specific inflammatory responses. Finally, the use of a more robust flow cytometry methodology that captures events related to apoptotic cell disassembly to analyze cell death allows us to have greater confidence in data generated (20), as conventional methodologies utilizing propidium iodide staining are suggested to have a false positive rate of up to 40% (59).

For this study, we used a submerged monolayer culture model that doesn't fully represent the physiological features of a differentiated respiratory epithelium (60). However, as the basal cells are epithelial progenitors, they are likely to represent intrinsic properties of the respiratory epithelium. Additionally, since viral replication and pro-inflammatory responses are elevated in air-liquid interface compared to monolayer culture (61) subtle phenotypic and mechanistic differences might be more easily identified in an air-liquid interface system. Rhinovirus species affect viral replication and inflammatory responses differently (17, 62), thus the implications of the findings from this study are limited to RV-A infections. However, RV-A has been reported as the most common strain present in adults with CF and associated with more severe clinical outcomes (63). We used a laboratory strain of RV-A (RV1b) that has been reported to induce cytotoxicity more readily than community-derived strains (8, 64, 65) and therefore future work will focus on corroborating the findings of this study using community RV strains of various serotype in order to determine if all RV induce inflammation via IL-1 signaling (66).

**Figure 5:**
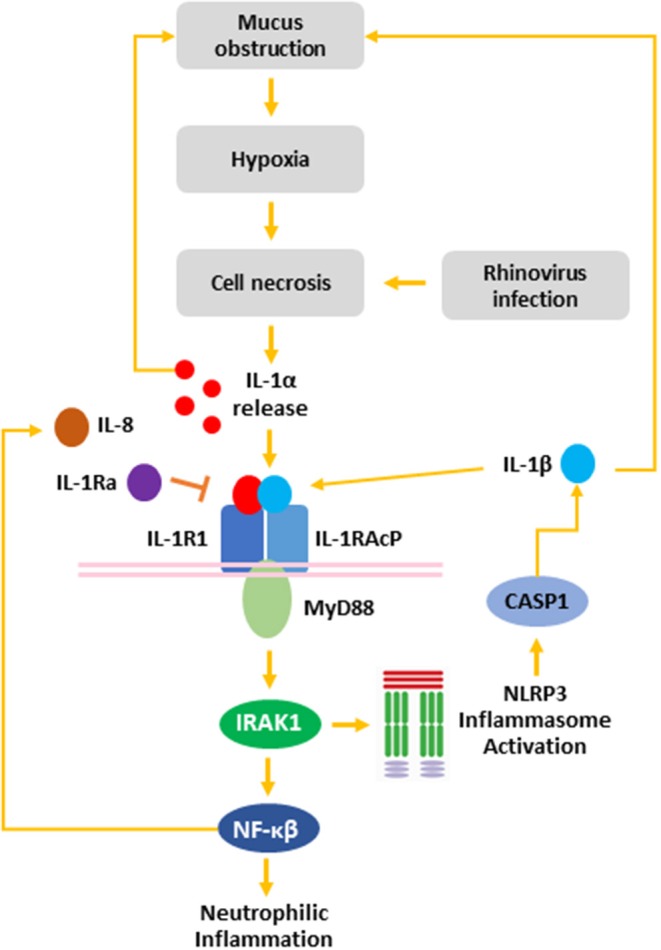
The role of rhinovirus infection in the IL-1 inflammatory response in the CF airway. Mucus obstruction in the CF airway leads to hypoxia of the airway epithelium and epithelial necrosis. Additionally, rhinovirus infection induced necrosis of AEC resulting in release of IL-1α from necrotic cells. Binding of IL-1α to IL-1R recruits MyD88 to the IL-1R:IL-1RAcP complex. Activation of MyD88 leads to IRAK1 activation, which activates the NLRP3 inflammasome leading to CASP1 activation and IL-1β secretion. Additionally, IRAK1 activates NF-κβ, which induces IL-8 release leading to neutrophilic airway inflammation. Both IL-1α and IL-1β induce mucin secretion, which leads to further mucus obstruction creating a positive feedback loop capable of exacerbating CF airway disease. IL-1R activation can be blocked by IL-1Ra to inhibit signaling downstream of IL-1R. IL-1α, interleukin-1 alpha; IL-1β, interleukin-1 beta; IL-1R1, interleukin-1 receptor 1; IL-1Ra, interleukin-1 receptor antagonist IL-1RAcP, interleukin-1 receptor accessory protein; IL-8, interleukin-8; IRAK, interleukin-1 receptor-activated protein kinase; MyD88, myeloid differentiation primary response gene 88; NFκB, nuclear factor kappa beta; NLRP3, nod-like receptor protein 3.

In summary, we have demonstrated that RV-A infection of non-CF and CF AEC drives necrotic cell death specifically associated with IL-1α and IL-1β in CF AEC. Viral infection also drove increased IL-8 release associated with necrotic cell death, implicating necrotic cell death following RV infection as a trigger of IL-1R-mediated neutrophilic inflammation in the CF airway. Collectively, these results suggest a role for RV infection as a trigger of IL-1R-driven neutrophilic inflammation in the early life CF airway (Figure 5). Mucin accumulation and hyperconcentration has been identified as the earliest trigger of cystic fibrosis lung disease (4), and linked to IL-1 signaling *in vitro* (43) creating a positive feedback cycle capable of inducing neutrophilic inflammation in the absence of bacterial infection. Previous studies have highlighted the potential translation of IL-1Ra as a novel anti-inflammatory therapy in CF (9, 12, 67, 68), with the aim to prevent further mucus obstruction and viral-induced, muco-inflammatory triggers of early lung disease in young CF children.

## Data Availability Statement

The raw data supporting the conclusions of this article will be made available by the authors, without undue reservation, to any qualified researcher.

## Ethics Statement

The studies involving human participants were reviewed and approved by The University of Western Australia Human Research Ethics Committee. Written informed consent to participate in this study was provided by the participants' legal guardian/next of kin.

## Author Contributions

SM, MM, SS, and AK contributed conception and design of the study. SM and DF acquired the data and performed the statistical analysis. SM, DF, MM, SS, and AK contributed to data analysis and interpretation. SM wrote the first draft of the manuscript. All authors contributed to manuscript revision, read, and approved the submitted version.

### Conflict of Interest

The authors declare that the research was conducted in the absence of any commercial or financial relationships that could be construed as a potential conflict of interest.
