# Supplementary material for: Rhinovirus Infection Is Associated With Airway Epithelial Cell Necrosis and Inflammation via Interleukin-1 in Young Children With Cystic Fibrosis
Source: Front Immunol. 2020 Apr 9;11:596. doi: 10.3389/fimmu.2020.00596 (PMC7161373; doi:10.3389/fimmu.2020.00596)
Supplement: Supplementary file 1 [file Data_Sheet_1.pdf]

Rhinovirus infection is associated with airway epithelial cell necrosis and inflammation via interleukin-1 in young children with cystic fibrosis

Samuel T. Montgomery, Dario L. Frey, Marcus A. Mall, Stephen M. Stick, Anthony Kicic on behalf of WAERP & AREST CF

Online Data Supplement

SUPPLEMENTAL METHODS

Study population

The AREST CF program covers a geographically defined population, with all children diagnosed in Western Australia managed at Perth Children's Hospital, Perth and children diagnosed in Victoria (excluding the south metropolitan area) are managed at Royal Children's Hospital, Melbourne. This study includes data from children diagnosed following detection by newborn screening since 2005, with >95% of eligible children participating. Written consent was obtained from each study participant's legal guardian after being fully informed about the premise and purpose of the study. Children with CF were recruited when they came to hospital for their annual early surveillance visit (1, 2). Healthy children were recruited during normal elective surgery for non-respiratory conditions and those with a pre-existing bacterial or viral chest infection were excluded from this study. The ISAAC and ATS respiratory questionnaires were used to confirm and validate the presence or absence of respiratory symptoms reported by parents or guardians.

Primary airway epithelial cell isolation and culture

Primary airway epithelial cells were isolated using either a trans-laryngeal non-bronchoscopy brushing method through an endotracheal tube, or a bronchoscopic brushing method through a bronchoscope (3). The non-bronchoscopic brushing was developed in the laboratory in which this study was performed and has been described previously (4, 5). Briefly, blind brushing was performed to sample bronchial airway epithelial cells. Each child was anaesthetized and intubated, before a cytology brush (BC 25105, Olympus, Australia) was inserted directly through the endotracheal tube, advanced until resistance was felt and rubbed in a circular motion against the epithelial surface to sample cells. The brush was then withdrawn and then cut off into in 4 mL cold RPMI-1640 and stored on ice. This brushing procedure was repeated with a second brush. Cell samples were immediately taken to the laboratory and processed within 15 minutes of sampling. After addition of 1 mL of FCS, epithelial cells were removed from the cytology brushes by vortex agitation. The brushes were placed in a new tube containing 4 mL of RPMI-1640 and 1 mL of FCS and vortex agitation was repeated to remove all cells. Collection media from both tubes were then pooled, centrifuged at 500g for 7 minutes at 4°C to pellet the cells and subsequently resuspended in Bronchial Epithelial Basal Medium (BEBM) (Lonza, Basel, Switzerland). To break up clumps of epithelia, the solution was passed three times through a 25G needle, before repeating with a 27G needle. The cell suspension was then incubated in a 35mm culture dish (BD Biosciences, Waltham, MA, USA) pre-coated with a 1:500 dilution of CD-68 antibody in 1x PBS for 20 minutes at 37°C in an atmosphere of 5% CO_2/95% air to remove any potential macrophages. Primary airway epithelial cells were then conditionally reprogrammed using a methodology optimised in this laboratory as previously described (6). Primary airway epithelial cells post-isolation were seeded at a density of 5000 cells/cm^2 into a culture vessel pre-coated with a fibronectin coating buffer containing γ-irradiated NIH-3T3 fibroblasts also seeded at a density of 5000 cells/cm^2. Primary airway epithelial cell cultures were maintained in an incubator at 37°C in an atmosphere of 5% CO_2/95%.

Rhinovirus production

Rhinovirus was produced utilising H1-HeLa cell culture as previously described (7). Briefly, viral stocks of crude RV1b were generated by infecting 5 x 10^7 cell/mL H1-HeLa cells in suspension culture with 2.5x10^8 $TCID_{50}$/mL RV1b and incubating for 8 hours at 35°C in an atmosphere of 5% CO_2/95% air. Cells were centrifuged at 500g for 7 minutes at 4°C to pellet the cells before resuspension in PBS with calcium and magnesium supplemented with bovine

albumin fraction V and storage at -80°C. Cells were disrupted by repeated freeze-thawing and the resultant suspension centrifuged at 10000g, before being aliquoted and stored at -80°C until use. Human rhinovirus titration was performed as previously described (8). Briefly, titration assays were performed by exposing confluent monolayers of MRC-5 cells in 48 well plates to serially diluted RV1b suspension in PBS with calcium and magnesium supplemented with bovine albumin fraction V ranging from 10^1-10^{15}. Initially, 50µL of virus suspension was added to each well for 1 hour to allow for virus attachment. Plates were then incubated in 0.5mL of the required growth medium for 14 days at 35°C in an atmosphere of 5% CO_2/95% air, and monitored at 1, 3, 5, 7, 9, 11 and 14 days for cytopathic effects. $TCID_{50}$/mL values were determined as previously described (8).

Quantification of inflammation

IL-1α, IL-1β, and IFN-β concentrations were determined in cell-free BAL and cell culture supernatants by a commercially available ALPHALisa kit (PerkinElmer, Waltham, MA) with working ranges between 1.3 and 30000pg/mL, 0.65 and 30000pg/mL, and 9.6 and 100000pg/mL respectively. Samples were measured in duplicate using an EnSpire 2300 plate reader (PerkinElmer, Waltham, MA). Data were crosstalk and blank-corrected and standard curves were fitted using 4-parameter logistic regression with data weighting.

IL-1Ra concentrations were determined in cell-free BAL and cell culture supernatants by a commercially available ELISA kit (DuoSet, R&D Systems, Minneapolis, MN, USA) with a working range between 39.1 and 2500pg/mL. sIL-1R2, CXCL10, CCL5, IL-28A, IL-28B, and IL-29 concentrations were determined in cell-free cell culture supernatants by commercially available ELISA kits (DuoSet, R&D Systems, Minneapolis, MN, USA) with working ranges outlined in **Table S2**. Samples were measured in duplicate using a BioTek SynergyMx (BioTek, Winnoski, VT, USA). Data were wavelength and blank-corrected and standard curves were fitted using 4-parameter logistic regression.

IL-8 concentrations were determined in cell-free BAL and cell culture supernatants by a commercially available ELISA kit (BD Opt EIA, BD Biosciences, San Diego, CA, USA) with a working range between 3.1 and 200pg/mL. Samples were measured in duplicate using a BioTek SynergyMx (BioTek, Winnoski, VT, USA). Data were blank and wavelength corrected, and standard curves were fitted using 4-parameter logistic regression.

SUPPLEMENTAL FIGURES

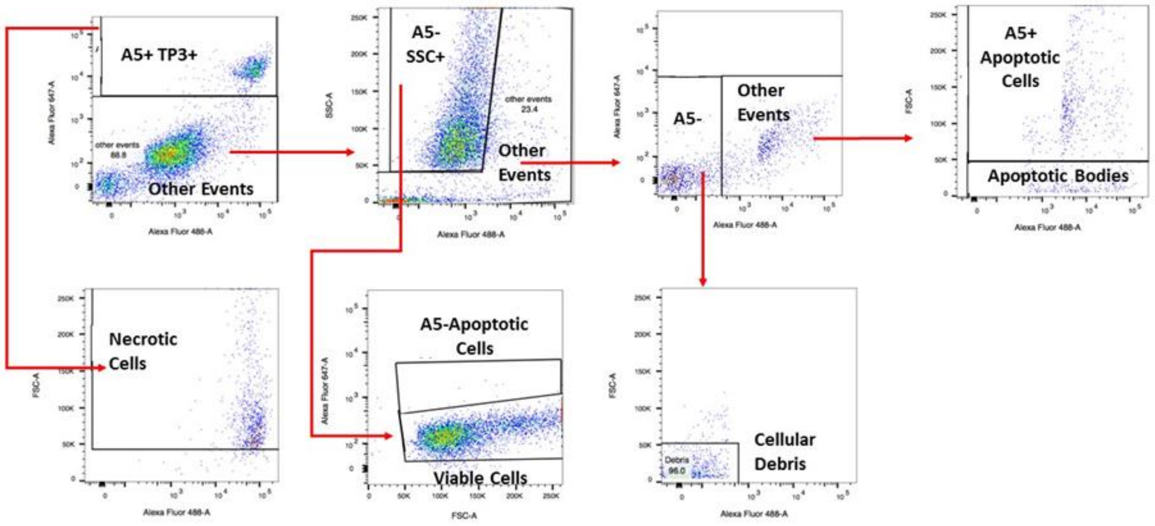

Figure S1: Representative gating strategy for flow cytometry analysis of AEC. Representative images for the flow cytometry analysis seven-stage electronic gating strategy utilised in this study to identify viable cells, A5- early apoptotic cells, A5+ early apoptotic cells, necrotic cells, apoptotic bodies and A5- particles/debris.

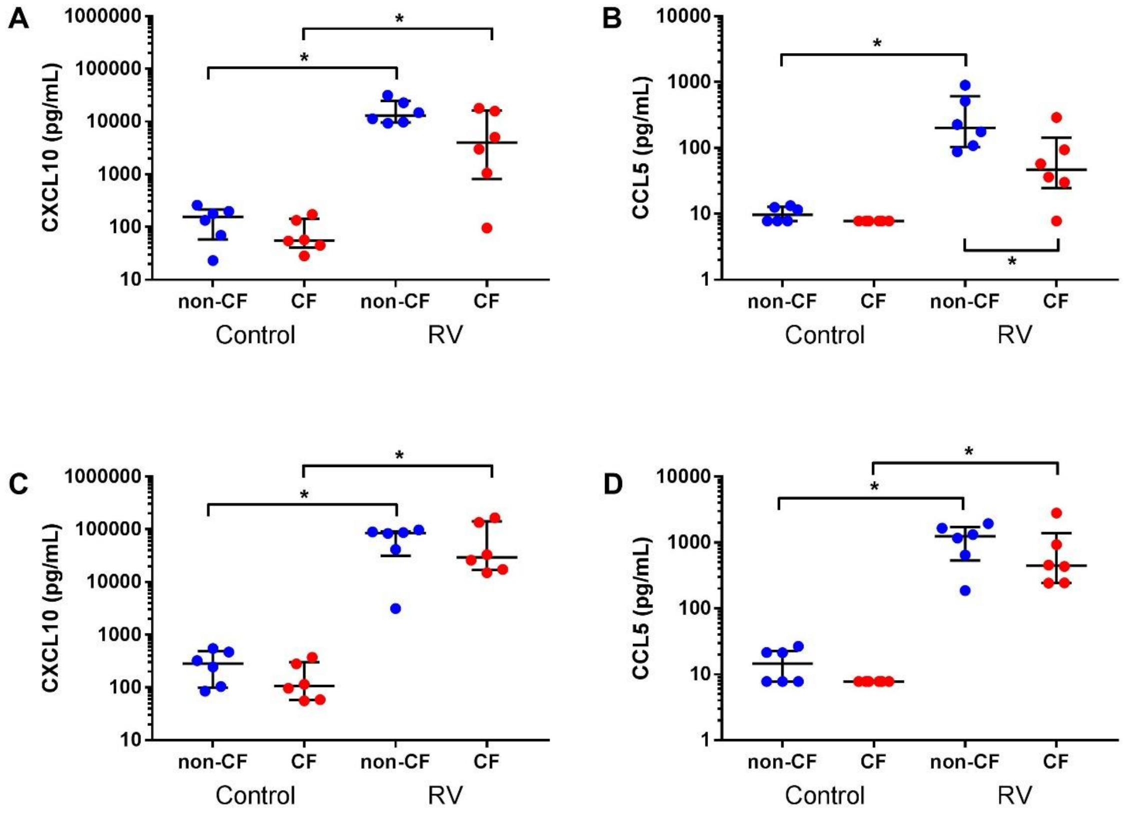

Figure S2: Rhinovirus infection increased CXCL10 and CCL5 protein in supernatant from non-CF and CF AEC. Supernatant from non-CF (n=6) and CF (n=6) AEC infected with RV1b for 24 and 48 hours were assessed for levels of CXCL10 and CCL5 protein. Infection with RV1b for 24 hours resulted in **(A)** increased CXCL10 in supernatant from non-CF and CF AEC compared to control, and **(B)** increased CCL5 in supernatant from non-CF and CF AEC compared to control, but significantly lower CCL5 in supernatant from CF AEC compared to non-CF AEC. Infection with RV1b for 48 hours resulting in **(C)** increased CXCL10 from non-CF and CF AEC compared to control, and **(D)** increased CCL5 from non-CF and CF AEC compared to control. *$p<0.05$.

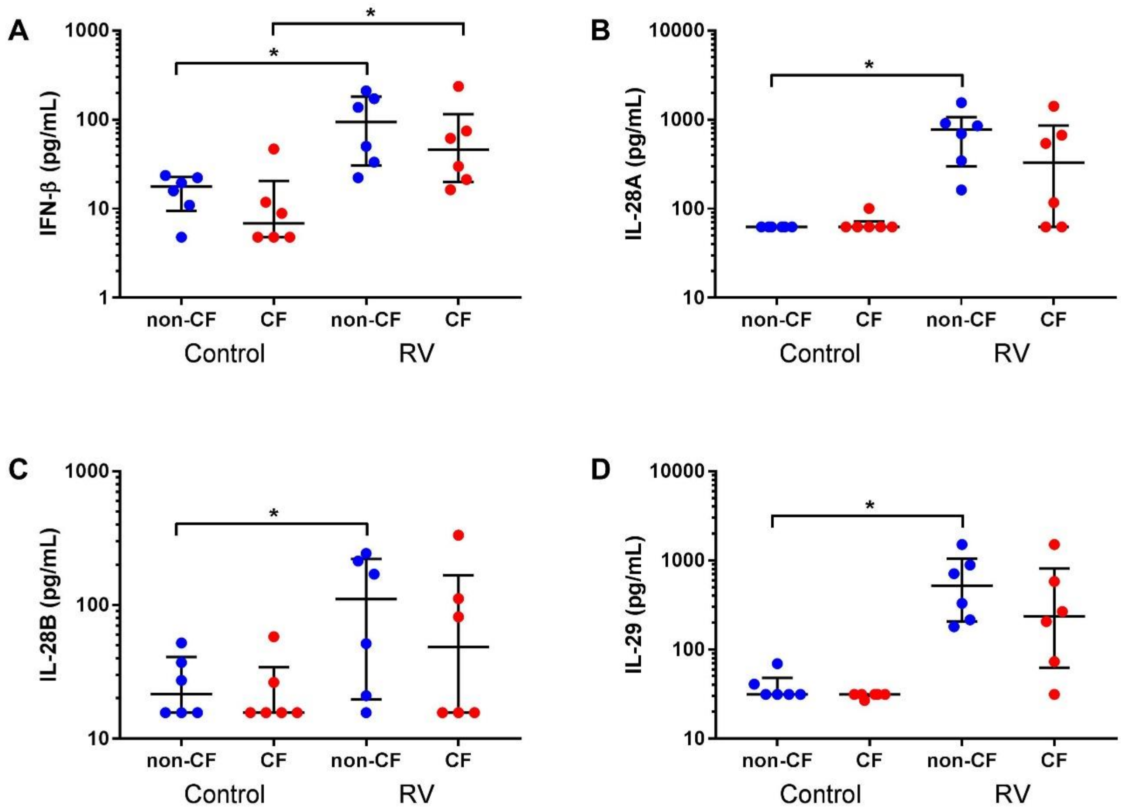

Figure S3: Interferon responses are increased following rhinovirus infection for 24 hours in non-CF AEC. Supernatant from non-CF (n=6) and CF (n=6) AEC infected with RV1b for 24 hours was assessed for levels of IFN-β, IL-28A, IL-28B, and IL-29 protein. Infection with RV1b for 24 hours resulted in **(A)** increased IFN-β in supernatant from non-CF and CF AEC compared to control, **(B)** increased IL-28A in supernatant from non-CF AEC but not CF AEC compared to control, **(C)** increased IL-28B in supernatant from non-CF AEC but not CF AEC compared to control, and **(D)** increased IL-29 in supernatant from non-CF AEC but not CF AEC compared to control. *$p<0.05$.

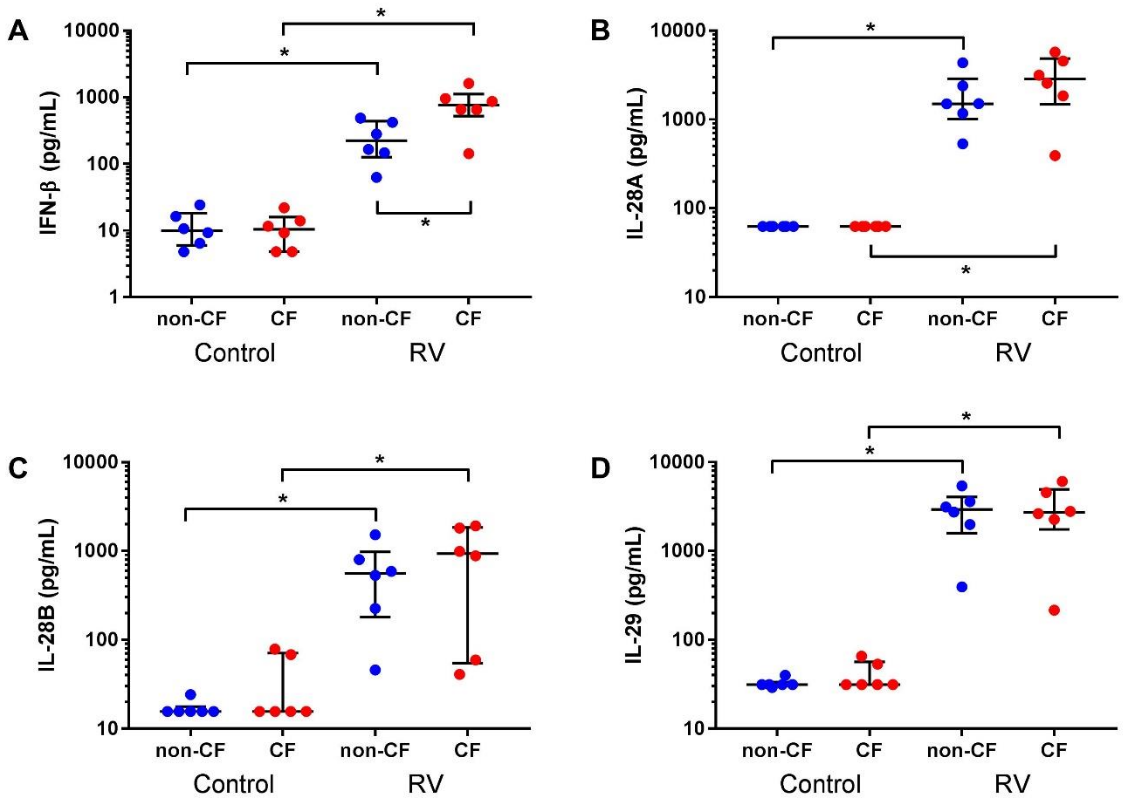

Figure S4: Interferon responses are increased following rhinovirus infection for 48 hours in non-CF and CF AEC. Supernatant from non-CF (n=6) and CF (n=6) AEC infected with RV1b for 48 hours was assessed for levels of IFN-β, IL-28A, IL-28B, and IL-29 protein. Infection with RV1b for 48 hours resulted in **(A)** increased IFN-β in supernatant from non-CF and CF AEC compared to control, with significantly higher IFN-β from CF AEC compared to non-CF AEC, **(B)** increased IL-28A from non-CF and CF AEC compared to control, **(C)** increased IL-28B from non-CF and CF AEC compared to control, and **(D)** increased IL-29 from non-CF and CF AEC compared to control. *$p<0.05$.

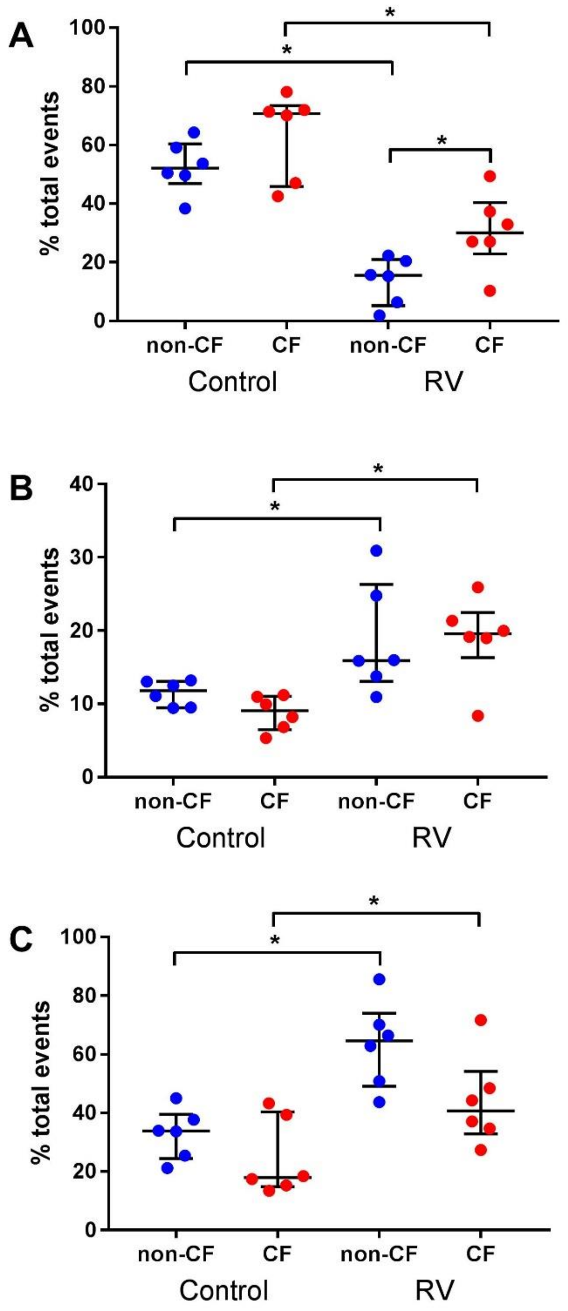

Figure S5: Rhinovirus infection of non-CF and CF AEC for 48 hours decreases viable events, increases necrotic events, and increases apoptotic events in non-CF AEC. Non-CF (n=6) and CF (n=6) AEC infected with RV1b for 48 hours were assessed for changes in viable **(A)**, necrotic **(B)**, and apoptotic **(C)** events measured via flow cytometry. Infection with RV1b for 48 hours resulted in **(A)** decreased viable events in non-CF and CF AEC compared to control, **(B)** increased necrotic events in non-CF and CF AEC compared to control, and **(C)** increased apoptotic events in non-CF and CF AEC compared to control. *$p<0.05$.

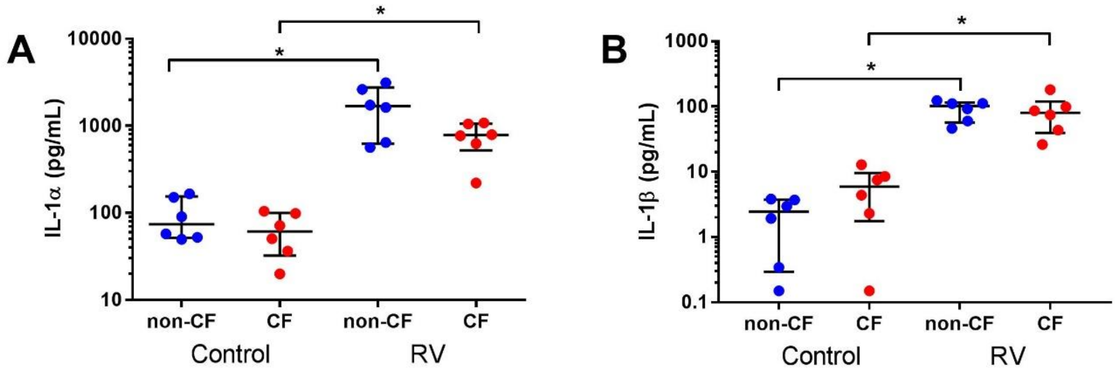

Figure S6: IL-1α and IL-1β is increased in supernatant from non-CF and CF AEC following 48 hours of rhinovirus infection. Supernatant from non-CF (n=6) and CF (n=6) AEC infected with RV1b for 48 hours was assessed for levels of IL-1α and IL-1β protein. Infection with RV1b for 48 hours **(A)** increased IL-1α from non-CF and CF AEC compared to control, and **(B)** increased IL-1β from non-CF and CF AEC compared to control. *$p<0.05$.

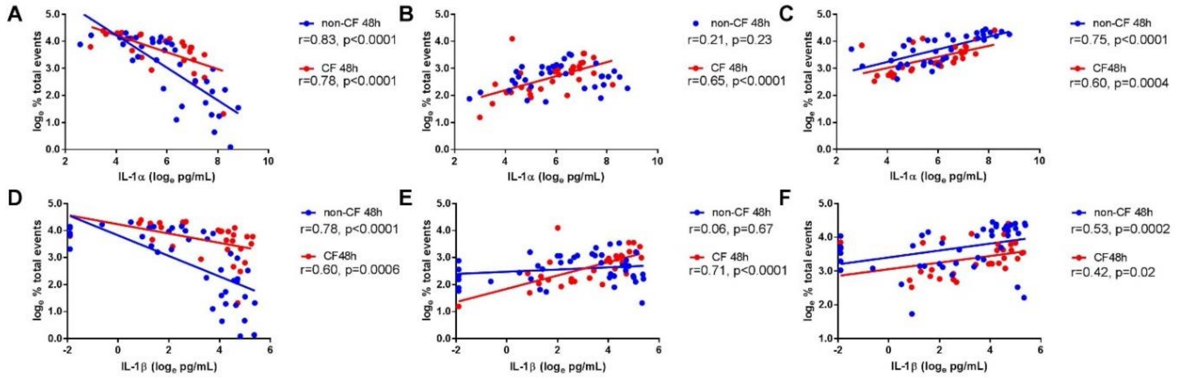

Figure S7: IL-1α and IL-1β in supernatant are associated with necrotic events in CF AEC but not non-CF AEC following 48 hours of rhinovirus infection. IL-1α and IL-1β protein in supernatant from non-CF and CF AEC following RV1b infection for 48 hours were assessed for correlations with the corresponding changes in viable, necrotic and apoptotic events measured via flow cytometry. IL-1α protein in supernatant was **(A)** significantly correlated with decreased viable events in non-CF and CF AEC, **(B)** significantly correlated with increased necrotic events in CF AEC but not non-CF AEC, and **(C)** significantly correlated with increased apoptotic events in non-CF and CF AEC. Similarly, IL-1β protein in supernatant was **(D)** significantly correlated with decreased viable events in non-CF and CF AEC, **(E)** significantly correlated with increased necrotic events in CF but not non-CF AEC, and **(F)** significantly correlated with increased apoptotic events in non-CF and CF AEC.

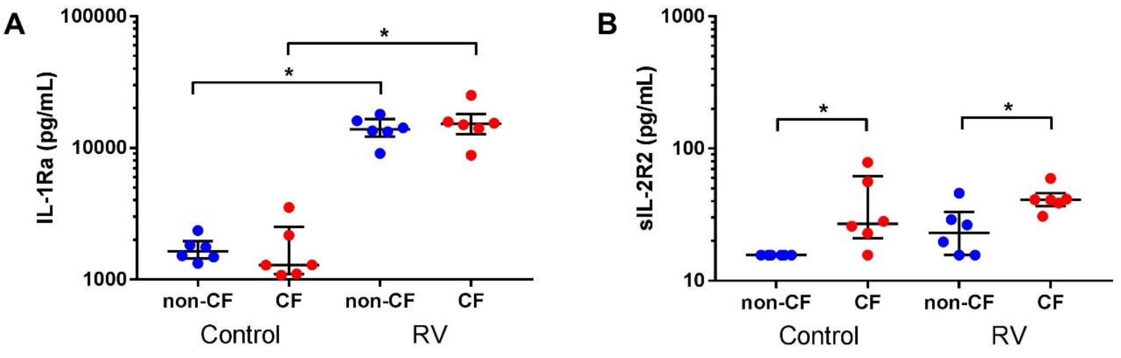

Figure S8: IL-1Ra but not sIL-1R2 is increased in supernatant from non-CF and CF AEC following 48 hours of rhinovirus infection. Supernatant from non-CF (n=6) and CF (n=6) AEC infected with RV1b for 48 hours was assessed for levels of IL-1α and IL-1β protein. Infection with RV1b for 48 hours **(A)** increased IL-1Ra from non-CF and CF AEC compared to control, with **(B)** no change in sIL-1R2 levels post-RV infection in non-CF and CF AEC compared to control, but higher sIL-R2 in CF AEC at baseline and following infection compared to non-CF AEC. *$p<0.05$.

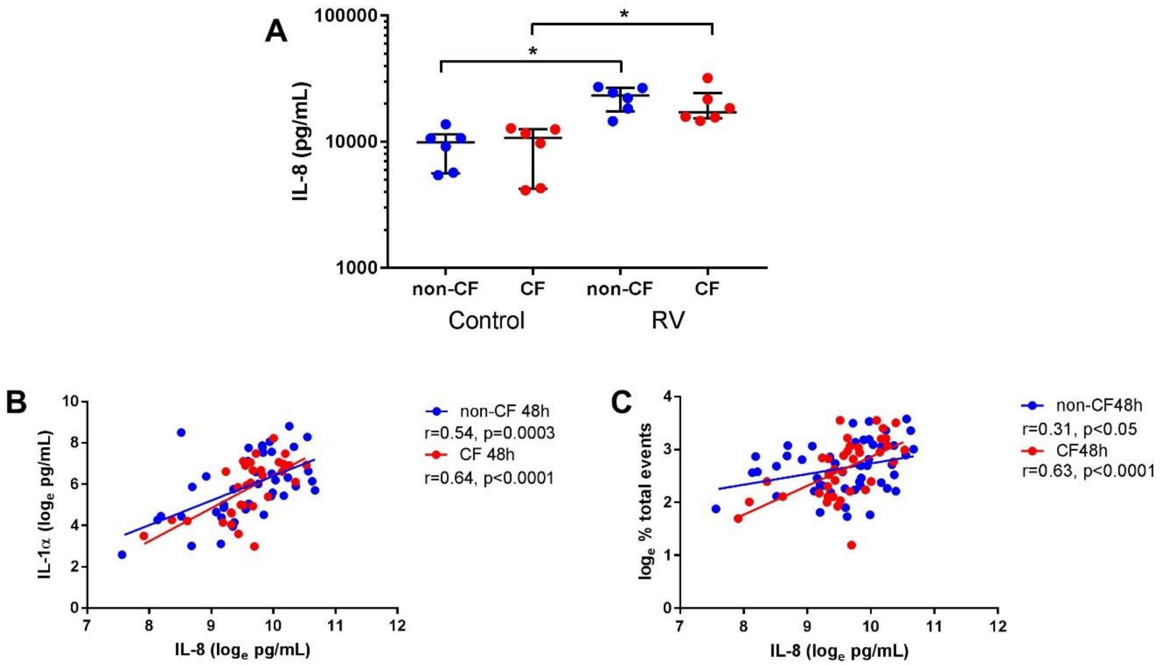

Figure S9: Rhinovirus infection for 48 hours increases IL-8 in supernatant from non-CF and CF AEC and associated with IL-1α and necrotic events. Supernatant from non-CF (n=6) and CF (n=6) AEC infected with RV1b for 48 hours was assessed for IL-8 levels. Infection with RV1b for 48 hours **(A)** increased IL-8 from non-CF and CF AEC compared to control, was **(B)** significantly positively correlated with levels of IL-1α in supernatant from non-CF and CF AEC, and **(C)** significantly positively correlated with necrotic events measured via flow cytometry in non-CF and CF AEC. *p<0.05.

SUPPLEMENTARY TABLES

	Non-CF		CF	
	**Control**	**RV1b**	**Control**	**RV1b**
**CXCL10**	144±86.9	**16539.1±8841.6**	82.0±57.9	**7141.3±7721.6**
**CCL5**	10.1±2.6	**334.2±314**	7.8±0	**85.7±103.6**
**IFN-β**	16.2±7.2	**104.1±79.6**	13.7±16.6	**73.5±83.4**
**IL-28A**	62.5±0	**753.2±488.7**	68.9±15.7	478.8±528.4
**IL-28B**	27.2±14.9	**119.1±101.8**	24.5±16.9	95.5±123.2
**IL-29**	39.2±15.2	**638.3±510**	30.6±1.8	444.4±557.2

Table S1: RV1b infection of AEC for 24 hours increases typical viral cytokine responses. Supernatant from non-CF and CF AEC infected with RV1b for 24 hours was assessed for CXCL10, CCL5, IFN-β, IL-28A, IL-28B, and IL-29. Data is presented as mean pg/mL ± standard deviation. Bold text indicates a significant difference between control and RV1b.

	Working Range (pg/mL	
Cytokine	Minimum	Maximum
**sIL-1R2**	31.2	2000
**CXCL10**	31.2	2000
**CCL5**	15.6	1000
**IL-28A**	125	8000
**IL-28B**	31.3	2000
**IL-29**	62.5	4000

Table S2: Working ranges of DuoSet ELISAs used for cytokine measurement.

REFERENCES

1. Stick SM, Brennan S, Murray C, Douglas T, von Ungern-Sternberg BS, Garratt LW, *et al.* Bronchiectasis in infants and preschool children diagnosed with cystic fibrosis after newborn screening. J Pediatr. 2009;155(5):623-8 e1.
2. Sly PD, Brennan S, Gangell C, de Klerk N, Murray C, Mott L, *et al.* Lung disease at diagnosis in infants with cystic fibrosis detected by newborn screening. Am J Respir Crit Care Med. 2009;180(2):146-52.
3. McNamara PS, Kicic A, Sutanto EN, Stevens PT, Stick SM. Comparison of techniques for obtaining lower airway epithelial cells from children. Eur Respir J. 2008;32(3):763-8.
4. Lane C, Burgess S, Kicic A, Knight D, Stick S. The use of non-bronchoscopic brushings to study the paediatric airway. Respir Res. 2005;6(1):53-.
5. Kicic A, Sutanto EN, Stevens PT, Knight DA, Stick SM. Intrinsic biochemical and functional differences in bronchial epithelial cells of children with asthma. Am J Respir Crit Care Med. 2006;174(10):1110-8.
6. Martinovich KM, Iosifidis T, Buckley AG, Looi K, Ling K-M, Sutanto EN, *et al.* Conditionally reprogrammed primary airway epithelial cells maintain morphology, lineage and disease specific functional characteristics. Scientific Reports. 2017;7(1):17971.
7. Lee W-M, Chen Y, Wang W, Mosser A. Growth of Human Rhinovirus in H1-HeLa Cell Suspension Culture and Purification of Virions. In: Jans DA, Ghildyal R, editors. Rhinoviruses: Methods and Protocols. New York, NY: Springer New York; 2015. p. 49-61.
8. Lee WM, Chen Y, Wang W, Mosser A. Infectivity assays of human rhinovirus-A and -B serotypes. Methods Mol Biol. 2015;1221:71-81.
